# Potential Impact of Physical Activity on Measures of Well-Being and Quality of Life in People with Rare Diseases: A Nationwide Cross-Sectional Study in Italy

**DOI:** 10.3390/healthcare12181822

**Published:** 2024-09-11

**Authors:** Silvana Mirella Aliberti, Anna Maria Sacco, Immacolata Belviso, Veronica Romano, Aldo Di Martino, Ettore Russo, Stefania Collet, Ilaria Ciancaleoni Bartoli, Manuel Tuzi, Mario Capunzo, Antonio Donato, Clotilde Castaldo, Franca Di Meglio, Daria Nurzynska

**Affiliations:** 1 Department of Medicine, Surgery and Dentistry “Scuola Medica Salernitana”, University of Salerno, 84081 Baronissi, Italy; sialiberti@unisa.it (S.M.A.); mcapunzo@unisa.it (M.C.); adonato@unisa.it (A.D.); dnurzynska@unisa.it (D.N.); 2 Department of Public Health, University of Naples “Federico II”, 80131 Naples, Italy; annamaria.sacco@unina.it (A.M.S.); immacolata.belviso@unina.it (I.B.); veronica.romano@unina.it (V.R.); aldo.dimartino@unina.it (A.D.M.); ettore.russo@unina.it (E.R.); manuel.tuzi@unina.it (M.T.); clotilde.castaldo@unina.it (C.C.); 3 Osservatorio Malattie Rare, 00187 Rome, Italy; stefania.collet@osservatoriomalattierare.it (S.C.); direttore@osservatoriomalattierare.it (I.C.B.)

**Keywords:** rare diseases, physical activity, well-being, quality of life, children-youth, adults, elderly

## Abstract

Background: Rare diseases constitute a heterogeneous group of approximately 7000–8000 conditions, distinguished by their low prevalence. Collectively, they present a significant global health challenge, affecting millions of people worldwide. It is estimated that rare diseases affect approximately 10% of the global population, which places a significant burden on individuals, families, and society. It is, therefore, important to consider strategies to improve the overall well-being and quality of life of individuals with rare diseases. One potential avenue for exploration is the incorporation of physical activity (PA). The scope of this study was to ascertain whether PA has a positive impact on measures of well-being and to determine its potential to enhance the quality of life of these individuals. Methods: The data were collected via an online survey. The one-way ANOVA test for multiple groups and multivariate Poisson models were employed to identify the significant predictors of the outcomes of interest. Results: The protective effects of PA become evident with a minimum of six hours of activity per week. Our data confirm that the weekly hours devoted to PA can serve as a significant protective factor for QoL. The study also provided some insights into the motivations behind patients’ engagement in PA. These included improving QoL and physical well-being, as well as the desire to interact socially, with the goal of meeting friends or making new acquaintances. Finally, for adults and older adults, engaging in PA can also be a way to control weight. Conclusions: It is becoming increasingly clear that individuals with rare diseases stand to benefit greatly from PA, so it is only sensible to educate them on the advantages of an active lifestyle.

## 1. Introduction

Rare diseases are a heterogeneous group of approximately 7000–8000 conditions, distinguished by their low prevalence, with an incidence of no more than 5 per 10,000 inhabitants in the European Union [1]. In the United States, rare diseases are defined as conditions affecting fewer than 200,000 people at any given time. Collectively, they present a significant global health challenge, affecting millions worldwide [2]. Globally, there are approximately 650 million individuals with disabilities, which represents roughly 10% of the world’s population [3].

It is estimated that approximately 80% of rare diseases have a genetic basis, while the remaining 20% are thought to arise from multifactorial causes [2], including individual susceptibility, environmental factors [4], and diet [5]. Some rare diseases result from the interplay between genetic and environmental factors. It is well established that environmental factors can induce a series of alterations in physiological development patterns through a process known as developmental plasticity, resulting in the production of alternative phenotypes throughout an individual’s lifespan. Such influence can have dual effects on health outcomes [6,7,8], as environmental factors can directly or indirectly modify the severity, timing, and presentation of the disease. This can occur through several mechanisms, including epigenetic influences, protein misfolding, alterations in miRNA activity, and regulation of mitochondrial function [4].

Despite their diversity, rare diseases exhibit common characteristics. Patients frequently encounter difficulties in obtaining a prompt and precise diagnosis. Additionally, there is a paucity of definitive treatments, and the diseases tend to be chronic and disabling. Consequently, rare diseases impose a significant burden on individuals, families, and society [1]. One strategy for enhancing the general well-being and promoting equitable opportunities in individuals afflicted with rare diseases is the incorporation of physical activity (PA) [9,10]. It has been demonstrated that engagement in PA can facilitate greater social integration [11], as it facilitates the establishment of social relationships. Furthermore, research has demonstrated that PA can confer psychological benefits, including improvements in self-esteem, autonomy, personal development, self-control, and self-confidence [11]. Sweeting et al. (2020) [12] posit that the practice of PA by individuals with rare diseases also yields physical benefits, including higher fitness levels, improved movement capacity, and enhanced functionality, ultimately promoting autonomy and self-sufficiency. In view of the evidence indicating that PA practice benefits this population of patients in both physical and mental health [13], it is imperative that they engage in sufficient levels of activity. Nevertheless, several studies have documented that individuals with disabilities engage in less PA than their healthy counterparts and lead a sedentary lifestyle [14,15]. These findings are consistent with those of Martin Ginis et al. (2021) [16], who have reported that individuals with rare diseases are between 16% and 62% less likely to achieve the recommended levels of PA and are at an elevated risk of developing health complications due to physical inactivity.

It is evident that to encourage the practice of PA among individuals with rare diseases, it is first essential to educate them on the advantages that an active lifestyle can offer. Secondly, it is of paramount importance to identify and address the potential obstacles that may impede the adoption of an active lifestyle with the aim of promoting health interventions [17]. This approach would allow for adapting PA provision to their motivations and minimising perceived barriers.

Despite the numerous studies on rare diseases that have been conducted to date, to the best of our knowledge, no research has been conducted to collect data at a national level, collecting data on 79 rare diseases with the objective of assessing the potential impact of PA.

In light of these considerations, the scope of our study was to ascertain whether PA has a positive impact on measures of well-being and to determine its potential to enhance the quality of life (QoL) of individuals with rare diseases.

## 2. Materials and Methods

### 2.1. Design and Sample of the Study

The study was designed and performed in accordance with the ethical principles set forth in the Declaration of Helsinki. In compliance with Legislative Decree 196/03 and the EU Regulation 2016/679 on the protection of personal data, the information contained was strictly personal and was addressed exclusively to the participants. All raw data were collected and stored by MT, who served as the legal responsible for the processing of the personal data, in accordance with Articles 28 and 39 of the GDPR.

The study employed a cross-sectional survey methodology and was conducted between June and October 2023 in all 20 Italian regions (with the exception of Valle D’Aosta, from which no response was received), with the objective of exploring the potential impact of PA on measures of well-being and QoL of children/youth, adults, and older adults with rare diseases. The data were collected from 397 patients, and their reported diseases were classified according to the World Health Organization’s (WHO) International Classification of Diseases (ICD-10) [18]. The various diseases were grouped based on their macrodomains. In our study group, 12 macrodomains were present (Table 1).

**Figure 1:**
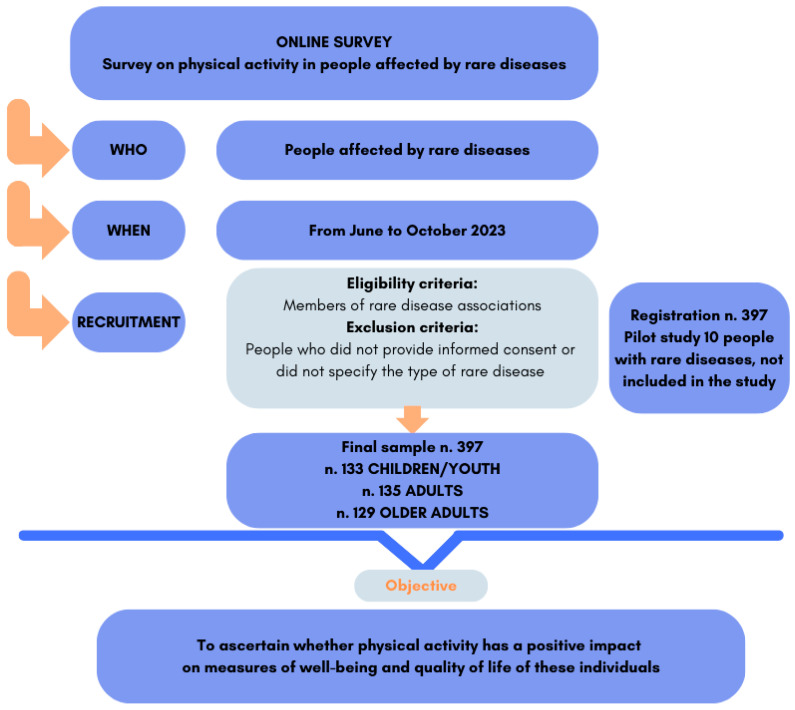
Depiction of the survey design, including the data collection, participant recruitment, and objective. For further details, please refer to the study design, instrument for collecting data, and results section.

The eligibility criteria for participation in the study included only individuals who were registered with Italian rare disease associations. The exclusion criteria included respondents to the questionnaire who did not provide informed consent or specify the type of rare disease (Figure 1).

The requisite sample size was determined through the application of the following equation [19]:

$$n=\frac{Z^{2}P\;(1-P)}{d^{2}}$$

In this equation, *n* represents the size of the sample, while *Z* is the *Z* statistic related to a specific confidence level. The term “*P*”, in this context, signifies the expected prevalence or proportion, while “*d*” is the precision associated with the sample. In our study, the *Z* value was calculated to be 1.96 for a 95% confidence level. The prevalence was determined to be 90% (in proportion to one) of patients who responded to the survey. The level of precision was estimated to be 3% (in proportion to one), and the recommended sample size was 384.

### 2.2. Procedure for Data Collection

The research team requested the assistance of rare disease associations in inviting their members to participate in the survey. The associations distributed the questionnaire through their websites and mailing lists.

The data were collected via a professional online survey platform (Google Forms), which provides several advantages. First, it offers an intuitive interface for data entry, which is convenient for respondents. Second, it provides audit trails to monitor data manipulation and export procedures, which ensures data integrity. Third, it offers automated export procedures for downloading data into common statistical packages, which saves time and resources. Finally, it provides procedures for importing data from external sources, which allows for the integration of diverse data sources.

The protocol guaranteed full anonymity, discretion regarding participation, and the absence of risk, conflict of interest, and incentives for participants. In order to ensure the anonymity of the respondents and facilitate the collection of self-reported data, only the socio-demographic items of age and place of residence were requested in the online survey. The participants expressed their consent before accessing the questionnaire via an electronic tool. Furthermore, to absolve the researchers of any liability, a text was included in the header of the web page with the questionnaire, which explained the objective of the study and the anonymous and voluntary nature of participation. It was requested that parents be present while minors completed the questionnaire.

### 2.3. Instrument for Collecting Data

The questionnaire was developed by the research team following two focus groups, during which the content was outlined. To ensure reliability and validity, the questionnaire was pretested with a random sample of 10 patients with rare diseases. Following the pretest, a few modifications were made to enhance the questionnaire’s comprehensibility. The results of the pilot study were not included in the final analysis (Figure 1). Following the pilot test, the research team approved the final version of the questionnaire.

The questionnaire consisted of four main sections: (1) socio-demographic characteristics of the respondent (age, region and municipality, pathology, whether he/she engages in PA). If the response to the question “Do you engage in PA?” was affirmative, the respondent proceeded to section (2), which included type of activity, duration, frequency, whether practised independently and reasons. If the response was negative, the respondent proceeded to section (3) of the questionnaire, which inquired about the reasons for not engaging in PA and the types of activities that were previously undertaken. Section (4) of the questionnaire addressed the availability of economic support for PA at the state, regional, provincial, and municipal levels, as well as the accessibility of appropriate facilities and qualified personnel. The questionnaire comprised a series of questions, including yes/no responses, open-ended questions, multiple responses, and four or five-point Likert scales.

### 2.4. Statistical Analysis

Descriptive statistics were used to summarise patient characteristics, and responses to all items were presented with absolute and relative frequencies for categorical variables. To compare variables within and between age groups, a one-way ANOVA test for multiple groups was used. Poisson regression analyses were performed to calculate the significant predictors of the measured variables across different age groups, disease macrocategories, and hours of PA per week. Incidence rate ratios (IRRs) and their 95% confidence intervals (CIs) were used in the Poisson regression models as measures of the independent associations between the different variables and the outcomes of interest. For all analyses, values equal to or less than 0.05 were regarded as statistically significant. Data analyses were performed with STATA software (release 16.1, StataCorp LLG, College Station, TX, USA, 2019).

## 3. Results

### 3.1. Socio-Demographic and Medical History Characteristics

The sample consisted of 397 patients with rare diseases, divided into three age groups. Of these, 133 belonged to the children/youth group, aged between 7–22 years; 135 belonged to the adult group, aged between 23–50 years; and 129 belonged to the older adults group, aged 51 years and older (Figure 1). The socio-demographic and anamnestic characteristics of the participants are reported in Table 2. The most frequent pathologies in our sample of patients with rare diseases were diseases of the nervous system (block G in ICD-10 classification), congenital malformations, deformations and chromosomal abnormalities (Q), diseases of the musculoskeletal system and connective tissue (M), diseases of the skin and subcutaneous tissue (L), followed by others listed in Table 2.

### 3.2. The Importance of PA between and within Age Groups

**Figure 2:**
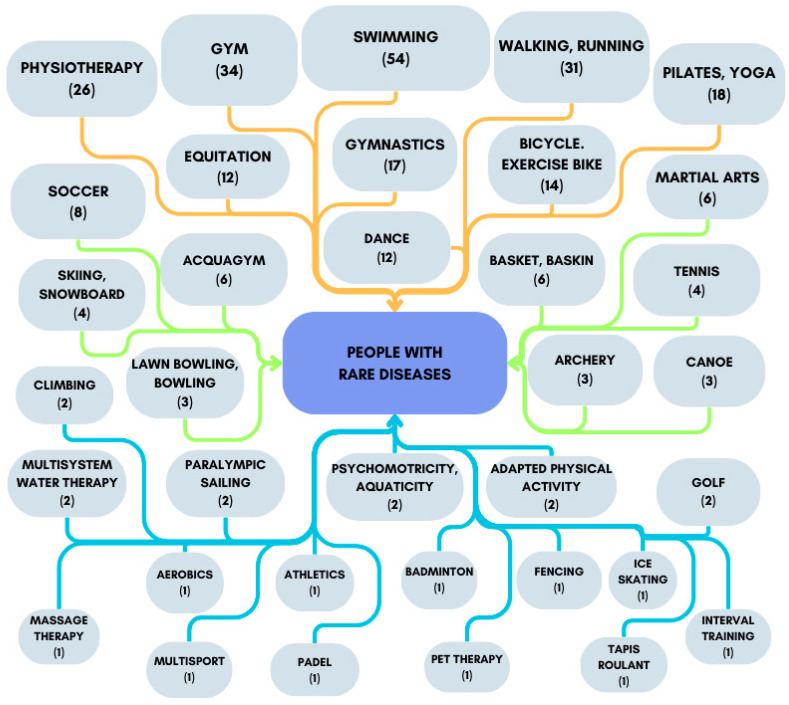
The graph presents an illustrative overview of the various types of PA (and the absolute frequency in parentheses) undertaken by people with rare diseases. The coloured arrows indicate the groupings of activities according to their higher or lower absolute frequencies. It should be noted that the number of each item may not correspond to the total number in the study population, as some participants engage in more than one activity.

Of the 397 patients in the sample, 205 engaged in PA. Of these, two-thirds practised one activity, while one-fourth practised two activities. The most common activities were swimming, gym training, walking or running, physical therapy, Pilates, cycling, gymnastics, dancing, and horseback riding (Figure 2). In the children and youth group, the period of PA ranged from one to 12 years, while in the adults and older adults groups, it ranged from one to 20 years. The weekly hours of PA also varied, with a mean of 3.7 ± 3 h. Importantly, children and youth carried out activities with the support of qualified staff, while adults and older adults participated independently.

A comparison of variables related to motivations for PA within and between age groups revealed that there was a statistical significance for weight control (*p* < 0.001) as well as for the following factors: engaging in social interaction with the goal of meeting friends or making new acquaintances (*p* = 0.04) and enhancing the physical condition (*p* = 0.005), which were also observed to have a significant impact on the quality of life (*p* = 0.04) (Table 3). The Poisson regression model, constructed to investigate the relationship between years of PA and the measure of well-being in the age groups, as well as the potential improvement of the disease, revealed that nine variables were statistically correlated with the outcome. These included age groups (IRR = 0.75, *p* < 0.001; IRR = 0.67, *p* < 0.001); endocrine, nutritional, and metabolic diseases (IRR = 0.78, *p* = 0.031); mental and behavioural disorders (IRR = 0.65, *p* < 0.001); congenital malformations, deformations and chromosomal abnormalities (IRR = 0.72, *p* = 0.001); diseases of the skin and subcutaneous tissue (IRR = 0.76, *p* = 0.008); diseases of the respiratory system (IRR = 0.49, *p* = 0.001); diseases of the blood and blood-forming organs and certain disorders involving the immune mechanism (IRR = 0.76, *p* = 0.016); and diseases of the nervous system (IRR = 0.67, *p* < 0.001) (Table 4, Model 1). Regarding the significant protective factors across disease macrocategories, findings suggest that engaging in at least six hours of PA per week may be beneficial (Table 4, Model 2). Furthermore, Poisson regression analysis demonstrated that the weekly hours of PA may serve as a significant protective factor for QoL (Table 4, Model 3).

### 3.3. The Reasons Why PA Is Not Practiced by Age Groups

Of the 397 patients in the sample, 192 did not engage in PA. The primary reasons for non-compliance were attributed to the following factors: (1) the presence of underlying medical conditions, (2) lack of qualified personnel and adequate facilities, (3) fatigue-related problems associated with the disease, and (4) cost of participation. Furthermore, individuals with disabilities also cited reasons such as “I have a disability”, “I don’t feel good about my body”, “I don’t feel like it”, “I lack the time to engage in leisure activities”, “My health is not optimal”, “I believe that physical activities are not necessary”, “I am too old”, “I am too young”, “I am ashamed of being seen doing it”, and “There is no provision for the activity I enjoy”.

### 3.4. The Evaluation of State or Local Support, Suitability of Facilities, and Trained Personnel for PA

Eighty percent of patients stated that the state, region, province, or municipality did not provide any support or financial assistance to encourage PA. Two-thirds of the total sample stated that there are no structures equipped for people suffering from pathologies, and if they exist, they have architectural barriers. Furthermore, 60% of those who do not carry out PA perceived that the facilities lack qualified personnel, while for 54% of those who do engage in PA, the qualified personnel at the facilities are too few. The importance of maintaining an active lifestyle is something that family doctors consistently emphasise, regardless of whether their patients engage or not in PA.

## 4. Discussion

The current study sought to ascertain the effect of PA on measures of well-being and QoL in a cohort of patients living with rare diseases. The protective effects of PA become evident with a minimum of six hours of activity per week. The study also provided some insights into the motivations behind patients’ engagement in PA. These included improving quality of life and physical well-being, as well as the desire to interact socially, with the goal of meeting friends or making new acquaintances. Additionally, for adults and older people, engaging in PA can also be a way to control weight.

PA is defined as any bodily movement produced by skeletal muscles that involves energy expenditure above the resting level [20]. This definition encompasses a wide range of activities, including household chores, work completed outside the home (professional activity), walking, bicycling, playing sports, and other activities of daily living or recreation [21].

It is widely recognised that PA offers a multitude of health and wellness benefits. These include improvements in body composition, prevention of overweight and obesity, and enhancement of skeletal, metabolic, and cardiovascular health [21]. In addition, there is growing evidence that PA has a positive impact on psychosocial well-being beyond its effects on biological health [22]. In fact, it can play a key role in improving general well-being and fostering equal opportunities for individuals with rare diseases [10]. In agreement with our results, it can be posited that the years of PA exert a potentially significant protective effect across all age groups. Furthermore, it was postulated that PA exerts a protective effect on rare diseases with significant implications for endocrine, nutritional, and metabolic diseases; mental and behavioural disorders; congenital malformations, deformations, and chromosomal abnormalities; diseases of the skin and subcutaneous tissue; diseases of the respiratory system; diseases of the blood and blood-forming organs and certain disorders involving the immune mechanism; and diseases of the nervous system.

The WHO recommends regular and adequate PA for all age groups, with specific guidance on the duration, intensity, frequency, and type of PA. For those unable to meet the recommendations for various health conditions, the WHO recommends that they engage in as much PA as possible [23]. The findings of our study indicate that the initiation of protective PA in rare diseases commences with the completion of six hours of activity per week. The most prevalent PA among the three age groups in our study were swimming, gym, walking or running, physiotherapy, Pilates or yoga, gymnastics, bicycling, and dance. A substantial body of epidemiological research indicates that PA plays a pivotal role in weight control. For instance, studies by Williamson et al. (1993) [24] utilising data from the National Health and Nutrition Examination Survey have demonstrated that low levels of self-reported recreational activities are associated with a heightened risk of major weight gain. This risk is three-fold higher in men and almost four-fold higher in women.

In analysing the significant protective factors across disease macrocategories, we determined that engaging in at least six hours of PA per week could be advantageous. The question of how much PA is needed to prevent weight gain has been the subject of much speculation. When being overweight is associated with rare diseases, the problem is even more pronounced. The American College of Sports Medicine (ACSM) position on this topic recommends 150–250 min per week of moderate to vigorous PA, with an energy equivalent of 1200 to 2000 kilocalories per week [25,26]. Saris et al. (2003) [27] proposed that individuals should engage in PA for 225–300 min per week to prevent the transition from normal weight to overweight or from overweight to obese. This aligns with the motivation of our adult and older adult participants to engage in PA for weight control.

The WHO offers the following definition of QoL: “an individual’s perception of their position in life in the context of the culture and value system in which they live and in relation to their goals, expectations, standards and concerns” [28]. It has been observed that the challenges associated with living with rare diseases can impact health-related QoL, that is, an individual’s perception of physical, mental, and social health [29,30]. Our study yielded some interesting insights into the motivations behind PA among patients. One of the primary findings was that a considerable number of patients were driven by a desire to enhance their QoL and physical well-being. Furthermore, the results of the study corroborated the hypothesis that the weekly hours of PA can serve as a significant protective factor for QoL.

Individuals affected with rare diseases frequently experience a dearth of social support and limited opportunities to engage in the typical social activities that contribute to a fulfilling life. This lack of social integration is often associated with social discrimination in various social contexts. However, several studies have identified social activity or social participation as a protective factor for the mental health [31,32,33] or the QoL [34] of those affected by rare diseases. In agreement with those findings, the primary motivation in our sample of children, youth, and older adults to engage in PA was the desire to interact socially, either by meeting friends or making new acquaintances.

A considerable body of research has demonstrated, however, that individuals with disabilities engage in significantly less PA than their healthy counterparts. Moreover, these studies observed a high prevalence of sedentary behaviour in people with illnesses [14,15]. In our study, just under half of the participants did not engage in PA, with many citing the disease as the reason for their sedentary lifestyle. A subsequent investigation was conducted to ascertain the primary reasons for this lack of PA. In addition to the underlying pathology, which prevents them from engaging in PA, or “easy tiredness” due to their illness, many patients cited reasons related to personal discomfort, such as “I don’t feel like it”, “I’m ashamed of being seen doing it”, “I don’t feel good about my body”. Consequently, the objective in the future is to educate patients suffering from rare diseases about the advantages of an active lifestyle while also promoting inclusiveness and acceptance from their healthy counterparts.

It is of critical importance to identify and address potential obstacles that could impede the adoption of an active lifestyle, with the aim of promoting effective health interventions [17]. The study revealed that patients perceive that their state, county, or municipality does not provide any support or assistance to encourage PA. Patients have to finance and provide for themselves in order to practice PA. In addition, there are no facilities equipped for people with disabilities, and when they do exist, they have architectural barriers. Other barriers included the lack or scarcity of qualified staff.

The significance of maintaining an active lifestyle was consistently emphasised by family doctors, regardless of whether patients engaged in physical exercise or not. This finding aligns with the assertion by Aliberti et al. (2022) [35] that family doctors play a pivotal role in monitoring the health status of the population. Such monitoring enables the early identification of potential deterioration in health, thereby reducing the financial burden on the National Health Service in terms of direct and indirect costs. It is, therefore, evident that doctors represent a key stakeholder group capable of providing a meticulous assessment of the general population’s health status, including the prevalence of specific diseases and the impact of various health issues. This assessment is particularly pertinent in the context of rare diseases, where the need for accurate data is paramount.

It should be noted that the present study has certain limitations. One of these is linked to the grouping of 79 rare diseases, which present a wide spectrum of different phenotypic manifestations and clearly differentiate patients in terms not only of basic clinical conditions but, above all, in terms of varying affinity to PA. It is, therefore, essential that these factors be investigated as much as possible in relation to the individual pathology. Other limitations of the study include the self-report method, which may be affected by recall bias or misreporting and the lack of potential depth. In light of the cross-sectional design of the present study, our future direction is to conduct a higher-level prospective study, which should allow for a more in-depth analysis of the cause–effect relationship.

## 5. Conclusions

In conclusion, it can be postulated that the overall duration of PA exerts a potentially significant protective effect in all age groups. Moreover, it has been proposed that PA has a protective effect in rare diseases, with significant implications for endocrine, nutritional and metabolic diseases; mental and behavioural disorders; congenital malformations, deformations, and chromosomal anomalies; diseases of the skin and subcutaneous tissue; respiratory system diseases; diseases of the blood and hematopoietic organs; and some disorders involving the immune mechanism; and diseases of the nervous system. The findings of our study indicate that this protective effect begins with engaging in six hours of activity per week. The study yielded some intriguing insights into the motivations behind PA among patients. One of the most significant findings was that a considerable number of patients were driven by a desire to improve their QoL and physical well-being. Our data substantiate the notion that weekly participation in PA can serve as a crucial protective factor for QoL. Furthermore, there was a desire to interact socially and meet friends or make new acquaintances. The motivation of the adult and older adults to engage in PA was also to control weight. General practitioners or family doctors play a fundamental role in monitoring the health status of the population.

In light of the beneficial effects of PA on individuals with rare diseases, it is evident that a crucial step in promoting its practice among this population is to educate them on the advantages of an active lifestyle. Additionally, it is vital to identify and address potential barriers to PA, with the objective of implementing effective health interventions.

## Figures and Tables

**Table 1:** Classification of rare diseases affecting people who participated in the survey, according to the WHO ICD-10 classification of diseases.

Disease Classification ICD-10		
Macrodomain	Block	Disease
Neoplasms	Malignant neoplasms	Hereditary Breast and Ovarian Cancer syndrome Epithelioid sarcoma Lymphoplasmacytic leukaemia
	Benign neoplasms	Lymphangioma
Diseases of the blood and blood-forming organs and certain disorders involving the immune mechanism	Coagulation defects, purpura, and other haemorrhagic conditions	Glanzmann disease Haemophilia
	Certain disorders involving the immune mechanism	Di George syndrome Sarcoidosis of lung
Endocrine, nutritional, and metabolic diseases	Metabolic disorders	Phenylketonuria Muscle carnitine palmityltransferase deficiency Medium-chain acyl-CoA dehydrogenase deficiency Homocystinuria Gaucher disease Renal tubulo-interstitial disorders in cystinosis Fabry disease Hunter syndrome Sanfilippo syndrome Acute porphyria Neuropathic heredofamilial amyloidosis Familial partial lipodystrophy
Mental and behavioural disorders	Disorders of psychological development	Autism Activity-dependent neuroprotective protein syndrome Rett syndrome
Diseases of the nervous system	Systemic atrophies primarily affecting the central nervous system	Friedreich ataxia (autosomal recessive) Ataxia telangiectasia Hereditary spastic paraplegy Spinal muscular atrophy
	Polyneuropathies and other disorders of the peripheral nervous system	Charcot-Marie-Tooth disease Hereditary neuropathy with liability to pressure palsies Chronic inflammatory demyelinating polyneuropathy Other polyneuropathy
	Diseases of the myoneural junction and muscle	Myasthenia gravis Myotonic dystrophy Nemaline myopathy Mitochondrial encephalomyopathy, lactic acidosis and stroke-like episodes Facioscapulohumeral muscular dystrophy
	Episodic and paroxysmal disorders	Dravet syndrome (severe myoclonic epilepsy of infancy) Ondine’s curse
	Other disorders of the nervous system	Myalgic encephalomyelitis
	Non-classified/non-specified	Cerebral angiopathy due to COL4A1 mutations, motor impairment, hemiparesis, epilepsy
Diseases of the eye and adnexa	Disorders of choroid and retina	Retinitis pigmentosa
Diseases of the circulatory system	Diseases of arteries, arterioles and capillaries	Raynaud syndrome
	Other forms of heart disease	Long QT syndrome Short QT syndrome
	Diseases of veins, lymphatic vessels and lymph nodes not elsewhere classified	Primary lymphoedema
Diseases of the respiratory system	Other respiratory diseases principally affecting the interstitium	Idiopathic pulmonary fibrosis
Diseases of the digestive system	Diseases of the oesophagus, stomach and duodenum	Achalasia of cardia
	Noninfective enteritis and colitis	Crohn disease Ulcerative colitis
	Diseases of liver	Hepatoportal sclerosis
	Non-classified/non-specified	Inflammatory bowel disease, short bowel syndrome, stoma carrier
Diseases of the skin and subcutaneous tissue	Atrophic disorders of the skin	Lichen sclerosus et atrophicus
	Urticaria and erythema	Urticaria due to cold and heat
Diseases of the musculoskeletal system and connective tissue	Inflammatory polyarthropathies	Still disease
	Systemic connective tissue disorders	Systemic lupus erythematosus Scleroderma Sjögren disease Behçet disease Antisynthetase syndrome
	Spondylopathies	Ankylosing spondylitis
	Soft tissue disorders	Fibromyalgia Myositis
	Inflammatory polyarthropathies	Psoriatic arthritis
Congenital malformations, deformations and chromosomal abnormalities	Congenital malformations and deformations of the musculoskeletal system	Macrodactylia Ehlers-Danlos syndrome
	Other congenital malformations	Epidermolysis bullosa Ectrodactyly-ectodermal dysplasia- clefting syndrome Neurofibromatosis CHARGE syndrome Alport syndrome Marfan syndrome Sotos syndrome Malan syndrome Prader–Willi syndrome

**Table 2:** Socio-demographic and anamnestic characteristics of respondents.

Characteristics of Respondents (Tot Sample = 397)	Doing PA (Tot Sample = 205)		Not doing PA (Tot Sample = 192)	
	N	%	N	%
Age in groups of year				
Children/Youth (7–22)	70	34.1	53	32.8
Adults (23–50)	78	48.1	57	29.7
Older adults (>50)	57	27.8	72	37.5
Region				
Piedmont	12	5.8	8	4.2
Liguria	5	2.4	1	0.5
Lombardy	41	20	33	17.2
Trentino-Alto Adige	2	0.9	-	-
Veneto	21	10.2	17	8.8
Friuli-Venezia Giulia	10	4.8	-	-
Emilia-Romagna	12	5.8	16	8.3
Tuscany	23	11.2	15	7.8
Umbria	3	1.5	-	-
Marche	-	-	4	0.2
Lazio	33	16.1	27	14.1
Abruzzo	8	3.9	6	0.3
Molise	1	0.5	-	-
Campania	12	5.8	35	18.2
Apulia	11	5.3	7	3.6
Basilicata	1	0.5	2	0.1
Sicily	1	0.5	7	3.6
Sardinia	7	3.4	8	4.2
Disease Macrodomains (WHO ICD-10 classification)				
Neoplasms (CD)	8	3.9	2	0.1
Endocrine, nutritional, and metabolic diseases (E)	11	5.3	15	7.8
Diseases of the digestive system (K)	5	2.4	8	4.2
Diseases of the eye and adnexa (H)	-	-	2	0.1
Mental and behavioural disorder (F)	13	6.3	13	6.8
Diseases of the circulatory system (I)	5	2.4	7	3.6
Congenital malformations, deformations and chromosomal abnormalities (Q)	33	16.1	22	11.4
Diseases of the musculoskeletal system and connective tissue (M)	23	11.2	22	11.4
Diseases of the skin and subcutaneous tissue (L)	23	11.2	18	9.3
Diseases of the respiratory system (J)	4	1.9	2	0.1
Diseases of the blood and blood-forming organs and certain disorders involving the immune mechanism (D)	15	7.3	12	6.2
Diseases of the nervous system (G)	63	30.7	65	33.8

**Table 3:** Comparison of variables related to motivations for PA within and between age groups.

What are the Motivations behind Engaging in PA?	Children/Youth			Adults			Older Adults			*p*
	No %	Enough%	Very%	No%	Enough%	Very%	No%	Enough%	Very%	
Improvement of disease problems	27.1	50	22.9	23.1	43.6	33.3	24.6	49.1	26.3	0.475
To control weight	57.1	38.6	4.3	28.2	50	21.8	28.1	59.6	12.3	<0.001
To engage in social interaction with the goal of meeting friends or make new acquaintances	28.5	58.6	12.9	51.3	35.9	12.8	43.8	52.7	3.5	0.046
To enhance the physical condition	14.3	61.4	24.3	5.1	48.7	46.2	3.5	63.2	33.3	0.005
To enhance mental well-being	11.4	61.4	27.2	11.5	46.2	42.3	1.8	61.4	36.8	0.159
To surmount obstacles	21.4	51.4	27.2	32.1	50	17.9	33.4	45.6	21	0.189
To enhance one’s quality of life	24.3	64.3	11.4	16.7	55.1	28.2	14	64.9	21.1	0.046
It was recommended by the attending physician	45.7	45.7	8.6	38.5	44.9	16.6	31.6	52.6	15.8	0.180

Note: *p*—*p* value.

**Table 4:** The results of a Poisson regression analysis of the outcomes of interest based on several explanatory variables.

Variable	IRR	95% CI	*p*
Model 1. Years of PA (Sample size = 203) Log likelihood = −779.1, x^2^ = 107.43 (12 df), *p* < 0.001			
Age in groups of years			
Children/Youth	1 ^a^		
Adults	0.75	0.68–0.83	<0.001
Older adults	0.67	0.59–0.75	<0.001
Disease Macrocategories			
CD	1 ^a^		
E	0.78	0.62–0.97	0.031
K	0.80	0.58–1.09	0.169
H	-	-	-
F	0.65	0.51–081	<0.001
I	0.91	0.69–1.19	0.523
Q	0.72	0.59–0.87	0.001
M	0.85	0.69–1.04	0.121
L	0.76	0.62–0.93	0.008
J	0.49	0.32–0.74	0.001
D	0.76	0.32–0.74	0.016
G	0.67	0.56–0.81	<0.001
Model 2. Disease Macrocategories (Sample size = 203) Log likelihood = −491.8, x^2^ = 19.55 (8 df), *p* = 0.012			
Age in groups of years			
Children/Youth	1 ^a^		
Adults	0.91	0.77–1.07	0.267
Older adults	0.88	0.74–1.04	0.146
Weekly hours of PA			
One hour	1 ^a^		
Two or two and a half hours weekly	0.91	0.73–1.14	0.447
Three hours weekly	0.87	0.69–1.09	0.237
Four hours weekly	1.01	0.80–1.28	0.911
Five hours weekly	0.87	0.65–1.15	0.334
Six hours weekly	0.58	0.41–0.84	0.004
>Of seven hours weekly	0.73	0.54–1.00	0.056
Model 3. Weekly hours of PA (Sample size = 205) Log likelihood = −392.7 x^2^ = 5.44 (1 df), *p* = 0.019			
Quality of Life	0.84	0.73–0.97	0.020

Note: IRR—incidence rate ratio; 95% CI—confidence interval; *p*—*p* value; ^a^—reference category; for further clarification regarding the disease acronyms presented in the table, please refer to Figure 1 or Table 1.

## Data Availability

For reasons of privacy, data cannot be shared with third-party organisations. The corresponding author is available to provide any explanation upon reasonable request.
